# The amino acid permease *SlAAP6* contributes to tomato growth and salt tolerance by mediating branched-chain amino acid transport

**DOI:** 10.1093/hr/uhae286

**Published:** 2024-10-11

**Authors:** Qi Qiang, Zhonghui Zhang, Xianggui Li, Chun Li, Mengdi Mao, Xiangyu Ding, Jianing Zhang, Shixuan Li, Zesen Lai, Jie Yang, Peng Cao, Weizhen Ye, Shouchuang Wang, Jun Yang

**Affiliations:** National Key Laboratory for Tropical Crop Breeding, School of Breeding and Multiplication Sanya Institute of Breeding and Multiplication, Hainan University, Sanya 572025, China; National Key Laboratory for Tropical Crop Breeding, College of Tropical Agriculture and Forestry, Hainan University, Sanya, Hainan 572025, China; National Key Laboratory for Tropical Crop Breeding, School of Breeding and Multiplication Sanya Institute of Breeding and Multiplication, Hainan University, Sanya 572025, China; National Key Laboratory for Tropical Crop Breeding, College of Tropical Agriculture and Forestry, Hainan University, Sanya, Hainan 572025, China; National Key Laboratory for Tropical Crop Breeding, School of Breeding and Multiplication Sanya Institute of Breeding and Multiplication, Hainan University, Sanya 572025, China; National Key Laboratory for Tropical Crop Breeding, College of Tropical Agriculture and Forestry, Hainan University, Sanya, Hainan 572025, China; National Key Laboratory for Tropical Crop Breeding, School of Breeding and Multiplication Sanya Institute of Breeding and Multiplication, Hainan University, Sanya 572025, China; National Key Laboratory for Tropical Crop Breeding, College of Tropical Agriculture and Forestry, Hainan University, Sanya, Hainan 572025, China; National Key Laboratory for Tropical Crop Breeding, School of Breeding and Multiplication Sanya Institute of Breeding and Multiplication, Hainan University, Sanya 572025, China; National Key Laboratory for Tropical Crop Breeding, College of Tropical Agriculture and Forestry, Hainan University, Sanya, Hainan 572025, China; National Key Laboratory for Tropical Crop Breeding, School of Breeding and Multiplication Sanya Institute of Breeding and Multiplication, Hainan University, Sanya 572025, China; National Key Laboratory for Tropical Crop Breeding, College of Tropical Agriculture and Forestry, Hainan University, Sanya, Hainan 572025, China; National Key Laboratory for Tropical Crop Breeding, School of Breeding and Multiplication Sanya Institute of Breeding and Multiplication, Hainan University, Sanya 572025, China; National Key Laboratory for Tropical Crop Breeding, College of Tropical Agriculture and Forestry, Hainan University, Sanya, Hainan 572025, China; National Key Laboratory for Tropical Crop Breeding, School of Breeding and Multiplication Sanya Institute of Breeding and Multiplication, Hainan University, Sanya 572025, China; National Key Laboratory for Tropical Crop Breeding, College of Tropical Agriculture and Forestry, Hainan University, Sanya, Hainan 572025, China; National Key Laboratory for Tropical Crop Breeding, School of Breeding and Multiplication Sanya Institute of Breeding and Multiplication, Hainan University, Sanya 572025, China; National Key Laboratory for Tropical Crop Breeding, College of Tropical Agriculture and Forestry, Hainan University, Sanya, Hainan 572025, China; National Key Laboratory for Tropical Crop Breeding, School of Breeding and Multiplication Sanya Institute of Breeding and Multiplication, Hainan University, Sanya 572025, China; National Key Laboratory for Tropical Crop Breeding, College of Tropical Agriculture and Forestry, Hainan University, Sanya, Hainan 572025, China; National Key Laboratory for Tropical Crop Breeding, School of Breeding and Multiplication Sanya Institute of Breeding and Multiplication, Hainan University, Sanya 572025, China; National Key Laboratory for Tropical Crop Breeding, College of Tropical Agriculture and Forestry, Hainan University, Sanya, Hainan 572025, China; National Key Laboratory for Tropical Crop Breeding, School of Breeding and Multiplication Sanya Institute of Breeding and Multiplication, Hainan University, Sanya 572025, China; National Key Laboratory for Tropical Crop Breeding, College of Tropical Agriculture and Forestry, Hainan University, Sanya, Hainan 572025, China; National Key Laboratory for Tropical Crop Breeding, School of Breeding and Multiplication Sanya Institute of Breeding and Multiplication, Hainan University, Sanya 572025, China; National Key Laboratory for Tropical Crop Breeding, College of Tropical Agriculture and Forestry, Hainan University, Sanya, Hainan 572025, China; Yazhouwan National Laboratory, Sanya, Hainan 572025, China; National Key Laboratory for Tropical Crop Breeding, School of Breeding and Multiplication Sanya Institute of Breeding and Multiplication, Hainan University, Sanya 572025, China; National Key Laboratory for Tropical Crop Breeding, College of Tropical Agriculture and Forestry, Hainan University, Sanya, Hainan 572025, China

## Abstract

Branched-chain amino acids (BCAAs) are essential amino acids in tomato (*Solanum lycopersicum*) required for protein synthesis, which also modulate growth and abiotic stress responses. To date, little is known about their uptake and transport in tomato especially under abiotic stress. Here, the tomato *amino acid permease 6* (*SlAAP6*) gene was identified as an amino acid transporter that restored mutant yeast cell growth on media with a variety of amino acids, including BCAAs. Overexpression of *SlAAP6* (*SlAAP6-OE*) in tomato raised the BCAA content and elevated the fresh weight, while *SlAAP6* knockouts (*slaap6*) showed reduced levels of neutral and basic amino acids in seedling tissues and lower total free amino acid distribution to shoots. In comparison to wild type and *slaap6* mutants, *SlAAP6-OE* alleviated root limited growth by elevated BCAA transport and upregulated the expression of root-growth-related genes by increasing BCAAs *in vivo*. As SlAAP6 serves as a positive regulator for BCAA abundance, *SlAAP6-OE* lines showed greater salinity tolerance, while *slaap6* mutants exhibited increased salt sensitivity. The salt tolerance of *SlAAP6-OE* plants was further enhanced by the application of exogenous BCAAs. In addition, BCAA supplementation reduced the accumulation of H_2_O_2_ in root under salt stress conditions. Based on these findings, SlAAP6-mediated uptake and transport of BCAAs facilitated growth and salt tolerance in tomato. By characterizing this key amino acid transporter, this study provides a novel approach to simultaneously enhance tomato nutritional quality, growth and development, and stress resistance through genetic improvement.

## Introduction

Tomato (*Solanum lycopersicum*) is an economically important horticultural crop that is particularly rich in metabolites, including essential amino acids, carotenoids, flavonoids, lipids, and terpenoids [1–7]. Branched-chain amino acids (BCAAs) are essential for plant growth and protein synthesis, necessitating their biosynthesis *in vivo* or intake from the environment [8, 9]. Over the long-term history of tomato domestication and improvement, the abundance of different amino acids in plant tissues has altered, resulting in complex agronomic traits [1, 10–12]. Thus, understanding how the genetic controls of BCAA biosynthesis will be essential for improving growth and development in tomato.

BCAAs, consisting of isoleucine (Ile), leucine (Leu), and valine (Val), are categorized by their branched hydrocarbon residues [13] and synthesized by four primary enzymes: acetohydroxyacid synthase, branched-chain aminotransferase, dihydroxyacid dehydratase, and ketol acid reductoisomerase [14–16]. When these enzymes are inactivated, a notable decrease in BCAA content ensues and root growth is inhibited [17, 18]. Some studies have demonstrated that amino acids can reduce the expression of *SHORT-ROOT* (*SHR*), a gene involved in the proliferation of root cells and regulation of cyclins; this reduction in *SHR* expression then impairs root development [19–22]. When the biosynthesis of Ile is interrupted, *AtCycB1* expression is altered, resulting in a lowered rate of cell division in the root apex [23]. Similarly, short-root phenotypes were observed in rice when BCAA accumulation was reduced [24]. In addition, many studies have illustrated BCAA accumulation in response to changing environmental stimuli, such as cold, drought, plant hormones, and salinity [25–28]. Decreasing BCAA content prevented root growth in NaCl treatments, but the growth of root was restored by exogenous BCAA supplementation [29–31]. This implies that BCAAs play an important role in mediating salt tolerance.

Amino acid transporters (AATs) are responsible for transporting and partitioning amino acids into different organs or tissues [32–34]. Most AATs facilitate biomass accumulation in plants and enhance stress resistance, especially when additional amino acids are supplied (e.g., as seen in *Arabidopsis* and rice). Amino acid permease (AAP) subfamily belongs to the amino acid/auxin permease (AAAP) family, which is one family of the AAT superfamily [35]. Overexpressing *OsAAP4* increased rice tillering and grain yield by increasing the abundance of the neutral amino acids proline (Pro) and threonine (Thr) [33]. In contrast, overexpressing *OsAAP3* or *OsAAP5* decreased tillering and grain yield as a result of enhancing the concentrations of the basic amino acids arginine (Arg) and lysine (Lys) [36, 37]. The lysine and histidine transporter AtLHT1 represents a key protein in the *Arabidopsis* defense response to *Hyaloperonospora arabidopsidis*; AtLHT1 promotes the accumulation of β-aminobutyric acid and R-β-homoserine [38–40]. While a comprehensive study identifying 88 AAT-encoding genes (grouped into 12 subfamilies) was recently conducted for tomato, only a few AATs have been functionally characterized [35]. For example, the proline transporter (ProT) subfamily protein LeProT1 supports tomato pollen development, germination, and subsequent pollen tube growth [41]. Cationic amino acid transporter 9 (SlCAT9), a tonoplast Glu/Asp/GABA exchanger, influences the accumulation of these amino acids during tomato fruit development [42]. Specific amino acids, Pro and BCAAs, can induce long-lasting stress resistance in plants [43, 44]. Some proline transporters, such as ProT1 and ProT 2, enhance plant resistance to salt stress by cellular import of L-proline, that as an osmoprotectant under stress treatment [45]. To date, the involvement of BCAA transporters in tomato growth and stress resistance remains incompletely characterized.

Here, 374 tomato accessions were analyzed in a metabolic genome-wide association study (mGWAS), and the *amino acid permease 6* (*SlAAP6*) gene was associated with BCAA content in this diverse tomato population. By further assessing gene expression patterns, overexpression lines, and knockout mutants to functionally characterize *SlAAP6*, it was found to support high BCAA levels in tomato seedlings. Overexpression of *SlAAP6* accelerated the accumulation of total free amino acids and promoted tomato growth and development through the alteration of BCAA uptake. Furthermore, *SlAAP6* conferred salinity tolerance in tomato by mediating BCAA transport, thereby supporting root elongation and reactive oxygen species (ROS) detoxification. The study findings provide novel insights into the crucial role of *SlAAP6* expression in mediating BCAA content to enhance tomato growth and salinity tolerance.

## Results

### *SlAAP6* is an amino acid transporter related to BCAA levels in tomato

**Figure 1:**
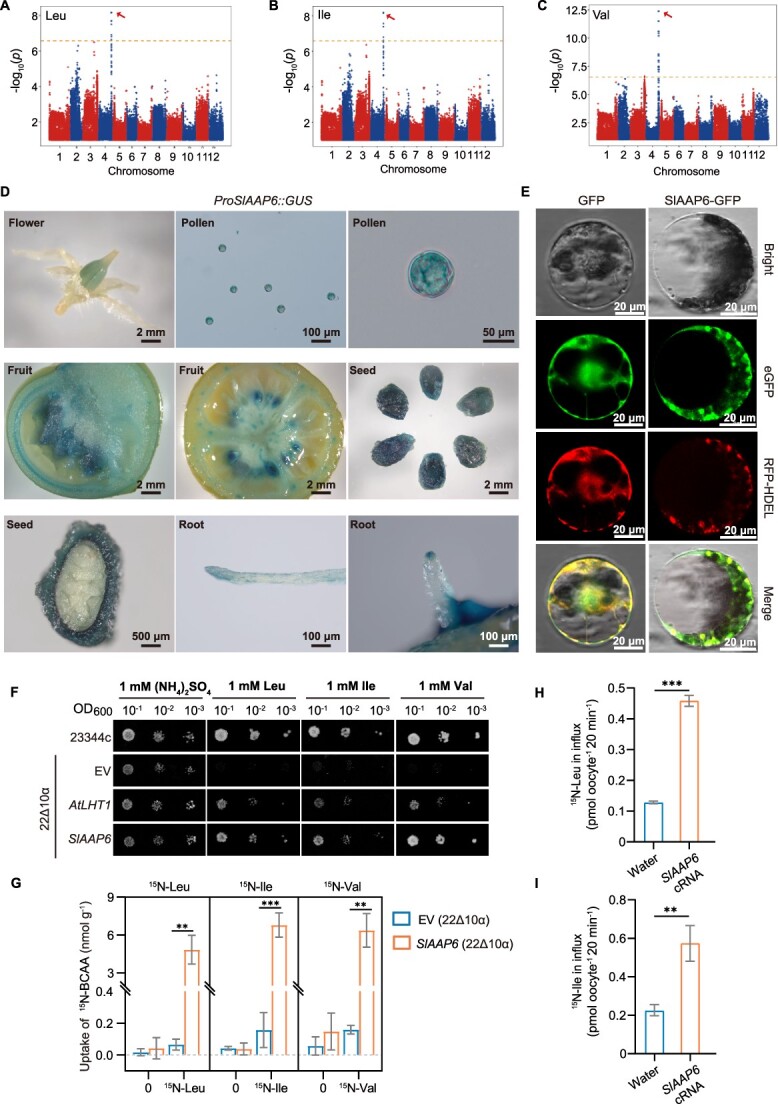
Identification and expression of the colocalized locus (*Solyc04g077050*) for determination of BCAA content in tomato accessions. **A–C**: Manhattan plots for leucine (Leu) (**A**), isoleucine (Ile) (**B**), and valine (Val) (**C**) mGWAS including 374 tomato accessions. **D**: GUS staining of transgenic seedlings expressing the *GUS* reporter gene with the *SlAAP6* promoter*.* From the first to the ninth photos (left to right, top to bottom), the GUS staining tissues were used from flower, pollen, fruit, seed, and root respectively. **E**: Subcellular localization analysis of SlAAP6 in protoplast. SlAAP6-GFP signals presented on ER and plasma membrane. The bar scale is 20 μm. **F**: Heterologous expression of *SlAAP6* in yeast strain 22Δ10α grown on 1 mM BCAAs, with a positive control (*AtLHT1*) and negative control (the empty pESC-URA vector, EV). The WT strain 23344c was also utilized as a growth control. **G**: Uptake of ^15^N-Leu, ^15^N-Ile, and ^15^N-Val was determined in 22Δ10α after feeding with ^15^N-Leu, ^15^N-Ile, ^15^N-Val (*n* = 3). **H–I**: The influx of ^15^N-Leu (**H**) and ^15^N-Ile (**I**) in *SlAAP6*-cRNA injected oocytes (*n* = 3). All values are means ± SDs. Significant differences (Student's *t*-tests): ^*^P < 0.05; ^**^P < 0.01; ^***^P < 0.001.

To characterize the genetic factors regulating BCAA content, a mGWAS was performed based on the content of Ile, Leu, and Val in the tomato fruit of 374 accessions from various subgroups, including 245 *S. lycopersicum* (BIG), 103 *S. lycopersicum var. cerasiforme* (CER), and 26 *Solanum pimpinellifolium* (PIM) [46]. The Manhattan plot presented significant single nucleotide polymorphism (SNP)-trait associations for the abundance of leucine (*P* = 6.69E^−09^), isoleucine (*P* = 6.64E^−09^), and valine (*P* = 3.91E^−13^) on chromosome 4 of a lead SNP (462032709) (Fig. 1A–C). Further analysis revealed that the SNP is in high linkage with the Solyc04g077050 gene and results in a nonsynonymous mutation in its coding sequence (CDS) where a G is mutated to T (Table S1). *Solyc04g077050* was annotated as an amino acid permease encoding a 482-amino acid protein named SlAAP6 for its high homology with AAP6 and AAP6-like proteins from *Arabidopsis* [47], *Brassica napus* [48], *Glycine max* [49], *Oryza sativa* [50], and *Solanum tuberosum* [51] (Fig. S1A).

To investigate the function of SlAAP6, tissue expression of GUS staining under control of the SlAAP6 promoter showed that *SlAAP6* was mainly expressed in tomato flowers, stems, roots, leaves, immature and breaker stage fruits, and seeds (Fig. S2), and especially in pollen, vascular fruit tissues, seed coats (at the reproductive stage), and roots (at the vegetative stage) (Fig. 1D). Additionally, transient expression of SlAAP6-GFP fusion protein was observed in both endoplasmic reticulum (ER) and plasma membrane in *Nicotiana benthamiana* protoplasts and transfected tobacco leave cells (Fig. 1E and Fig. S3). Consistent with bioinformatic predictions of 11 transmembrane domains within SlAAP6 (Fig. S1B), the protein was localized at ER membrane and plasma membrane, indicating that SlAAP6 plays an important role in membrane system.

To verify the role of SlAAP6 in BCAA transport, *SlAAP6* was transformed into yeast mutant strain 22Δ10α [52], which is thoroughly deficient in the uptake of proteinogenic amino acids and unable to grow on medium containing amino acids. Heterologous expression of *SlAAP6* rescued 22Δ10α growth when the growth medium included Ile, Leu, or Val, indicating that SlAAP6 transported BCAAs (Fig. 1F). Additionally, *SlAAP6* expression also allowed 22Δ10α growth on media containing alanine, phenylalanine, proline, threonine, or tyrosine (Ala, Phe, Pro, Thr, or Tyr) (Fig. S4). Furthermore, ^15^N-BCAAs were used as substrates to feed the mutant yeast strain, resulting in a significant higher accumulation of labeled BCAAs in yeast due to SlAAP6 transport (Fig. 1G). Moreover, *Xenopus* oocytes transport assay was performed to further confirm the transport of amino acids by SlAAP6. The oocytes were injected with *SlAAP6*-cRNA or water (control) and then subjected to ^15^N-labeled Leu and Ile transport analysis after 24 h. When incubated in an ND96 medium containing ^15^N-labeled Leu and Ile, the ^15^N-labeled tracer influx of cRNA-injected oocytes was higher than that of control oocytes (Fig. 1H and I). Therefore, SlAAP6 is responsible for transporting a range of amino acids.

### SlAAP6 enhances amino acids absorption and redistribution

**Figure 2:**
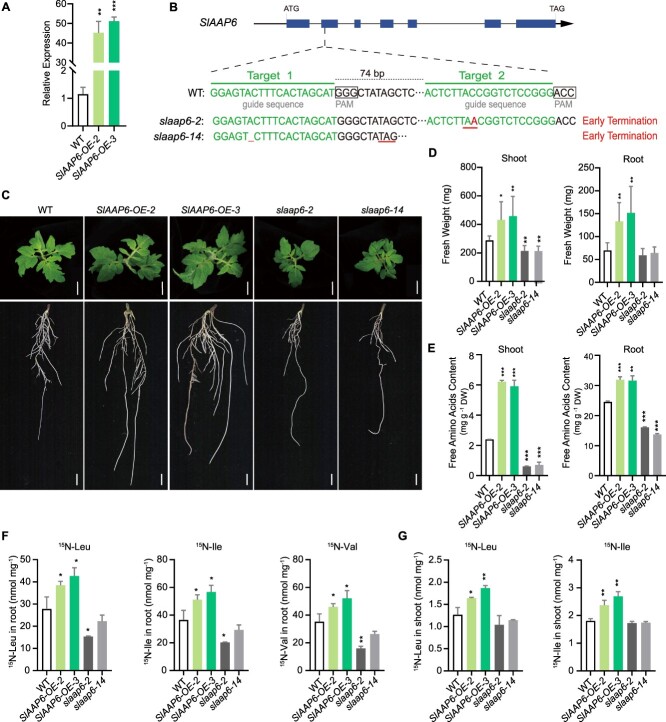
Phenotypic comparison between *SlAAP6-OE* lines and *slaap6* mutants. **A**: Relative expression of *SlAAP6* in WT, *SlAAP6-OE-2*, and *SlAAP6-OE-3*. **B**: Design of the knockout target sites for *SlAAP6* (*slaap6*) and sequence information for early translation termination in transgenic materials. The target CRISPR/Cas9 sequence is in the second exon of *SlAAP6*. **C**: Growth comparison of aerial parts and roots in 21-day-old tomato seedlings of WT and *SlAAP6* transgenic plants. Scale bar, 1 cm. **D**: Fresh weight of WT and *SlAAP6* transgenic plants (*n* = 6). **E**: Quantification of total free amino acid content in WT and *SlAAP6* transgenic plants (*n* = 3). DW, dry weight. **F**: Accumulation of ^15^N-labeled BCAAs in root of WT and *SlAAP6* transgenic plants (*n* = 3). **G**: Accumulation of ^15^N-Leu and ^15^N-Ile in shoot of WT and *SlAAP6* transgenic plants (*n* = 3). All values are means ± SDs. Significant differences (Student's *t*-tests): ^*^P < 0.05; ^**^P < 0.01; ^***^P < 0.001.

To explore the physiological function of the SlAAP6 transporter in tomato plants, *SlAAP6* overexpression (*SlAAP6-OE*) and *SlAAP6* knockouts (*slaap6*) transgenic lines were generated (Fig. 2A and B). Two mutants, *slaap6-2* and *slaap6-14*, had a 1-bp mutation and deletion at the second and first desired target sites, respectively, leading to early termination of SlAAP6 translation (Fig. 2B). As the transgenic tomato seedlings developed, *SlAAP6-OE* lines showed significantly higher shoot and root fresh weight compared to wild type (WT) at 21-day-old seedlings stage, whereas the *slaap6* mutants exhibited the opposite phenotype (Fig. 2C and D). Although SlAAP6 transported eight amino acids in mutant yeast strain, its effect on modulating of amino acid content in tomato remains unknown. Hence, ninhydrin colorimetry was used to determinate total free amino acid content in both aerial and underground parts of WT and *SlAAP6-*transgenic lines. The total free amino acid content was higher in *SlAAP6-OE* lines compared to the WT (in both above- and below-ground tissues), and lower in *slaap6* mutants (Fig. 2E). The contents of total nitrogen (N), which is a crucial determinant of plant growth, were detected through Kjeldahl method. The result showed that total N were increased more greatly and in whole seedlings of *SlAAP6-OE* than in those of WT, indicating that overexpression of *SlAAP6* enhanced total amino acid and nitrogen accumulation in whole plantlets (Fig. S5). These findings illustrate that *SlAAP6-OE* facilitated biomass accumulation, amino acid content, and nitrogen import in whole tomato seedlings.

Since *SlAAP6* was expressed in the vascular tissues and root rhizodermis, the effects of SlAAP6 on the uptake and distribution of individual amino acids were examined through liquid chromatography–mass spectrometry (LC–MS). Compared to the WT, the Ile (42%), Leu (40%), and Val (52%) contents were significantly higher in *SlAAP6-OE* lines, whereas the opposite was true in *slaap6* mutants (Fig. S6A–C). Basic and part of neutral amino acids were also elevated in *SlAAP6-OE* lines, but not in *slaap6* (Fig. S6). To analyze whether SlAAP6 plays a role in the allocation of BCAAs from root to shoot, the roots of WT and *SlAAP6*-transgenic lines were exposed to the solution containing ^15^N-Leu, ^15^N-Ile, or ^15^N-Val for 6 h to facilitate root-to-shoot ^15^N translocation. The results presented that the accumulation of all ^15^N-labeled BCAAs in roots of *SlAAP6-OE* lines were notably higher than that of WT, whereas the content was low in *slaap6* mutants (Fig. 2F). To examine SlAAP6 role in BCAA allocation, shoot samples of transgenic lines were also collected to determine. In contrast, ^15^N-Leu, ^15^N-Ile, and ^15^N-Val showed elevated content in *SlAAP6-OE* lines compared to WT, but ^15^N-Val was not transported to shoot in *slaap6* mutants (Fig. 2G and Table S2). Thus, the SlAAP6 protein facilitates the uptake of amino acids by the roots and subsequently transports them from the roots to the aerial parts.

### SlAAP6 modulates tomato root growth under BCAA supplementation

**Figure 3:**
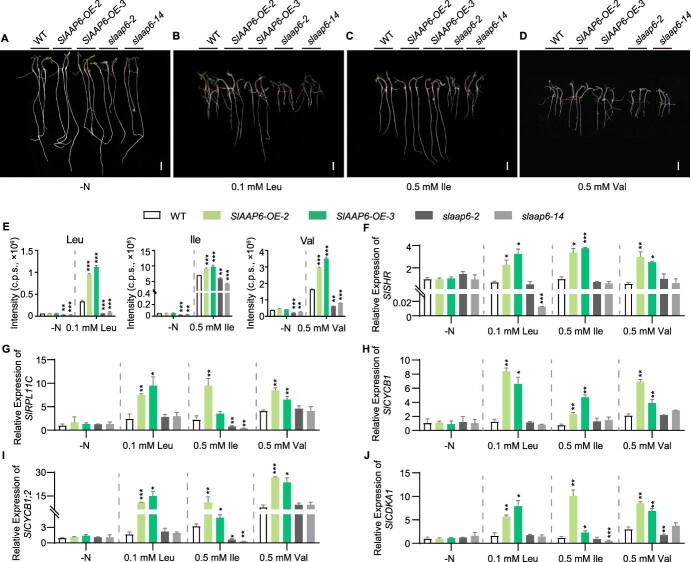
The impact of exogenous BCAA application on root growth in *SlAAP6-OE* and *slaap6* mutants. **A–D**: Root phenotypes in WT, *SlAAP6-OE-2*, *SlAAP6-OE-3*, *slaap6-2*, and *slaap6-14* lines grown without nitrogen (**A**), with 0.1 mM Leu (**B**), with 0.5 mM Ile (**C**), or with 0.5 mM Val (**D**). Scale bars, 1 cm. **E**: The relative content of Leu, Ile, and Val in WT and *SlAAP6* transgenic plants grown without nitrogen (**A**), with 0.1 mM Leu (**B**), with 0.5 mM Ile (**C**), or with 0.5 mM Val (**D**) (*n* = 3). **F–J**: The relative expression of *SlSHR* (**F**)*, SlRPL11C* (**G**)*, SlCYCB1* (**H**)*, SlCYCB1;2* (**I**), and *SlCDKA1* (**J**) in WT and *SlAAP6* transgenic plants grown without nitrogen, with 0.1 mM Leu, with 0.5 mM Ile, or with 0.5 mM Val (*n* = 3). All values are means ± SDs. Significant differences (Student's *t*-tests): ^*^P < 0.05; ^**^P < 0.01; ^***^P < 0.001.

As SlAAP6 accelerates root uptake and allocation of BCAAs, the role of SlAAP6 on root growth under BCAA treatments was further to verify. Studies in other species have shown that plants exposed to high concentrations of individual amino acids present reduced growth phenotypes [53, 54]. To determine the role of BCAAs in tomato growth and development, tomato seedlings were cultivated on media containing a range of concentrations (i.e., 0, 0.1, 0.5, or 1 mM) of Ile, Leu, or Val as the sole N source. There was no observation of a significant difference in the growth of WT and transgenic seedlings under the N-deficient treatment (Fig. 3A). However, a gradual inhibition of root growth was observed in WT and *slaap6* seedlings when the Ile, Leu, or Val concentration was 0.1 mM or higher, but *SlAAP6-OE* lines exhibited milder inhibitory (Fig. 3B–D and Fig. S7). This suggests that overexpression of *SlAAP6* in seedling roots conferred tolerance to exogenous BCAA application compared to WT roots, resulting in differences in root growth between transgenic plants and the WT. The reduced root growth observed here might be attributed to differences in BCAA transport between WT and transgenic seedlings. To test this hypothesis, optimal concentrations of Ile (0.5 mM), Leu (0.1 mM), and Val (0.5 mM) were selected for further investigation (Fig. 3A–D), as these concentrations produced the most pronounced differences in root length among WT plants, *SlAAP6-OE* lines, and *slaap6* mutants (Fig. S7). At these media concentrations, the Ile, Leu, or Val content was higher in the *SlAAP6-OE* lines versus the WT, and lower in the *slaap6* mutants (Fig. 3E), indicating that SlAAP6 could mitigate reduced growth caused by high amino acid concentrations through facilitating the translocation of BCAAs into plants.

To investigate how high BCAA levels affected cell division under BCAA application (Fig. 3E), the expression of root development- and proliferation-related genes was also examined. The expression of cell division protein kinase (*SlCDKA1*), cyclin-b (*SlCYCB1*, *SlCYCB1;2*), ribosomal protein L11C (*SlRPL11C*), and *SlSHR* was upregulated in *SlAAP6-OE* roots as compared to the WT (Fig. 3F–J). Therefore, these findings demonstrate that *SlAAP6-OE* promoted root growth by enhancing BCAA transport, leading to greater expression of genes associated with root elongation.

### *SlAAP6* promotes BCAA uptake and transport to resist saline stress

**Figure 4:**
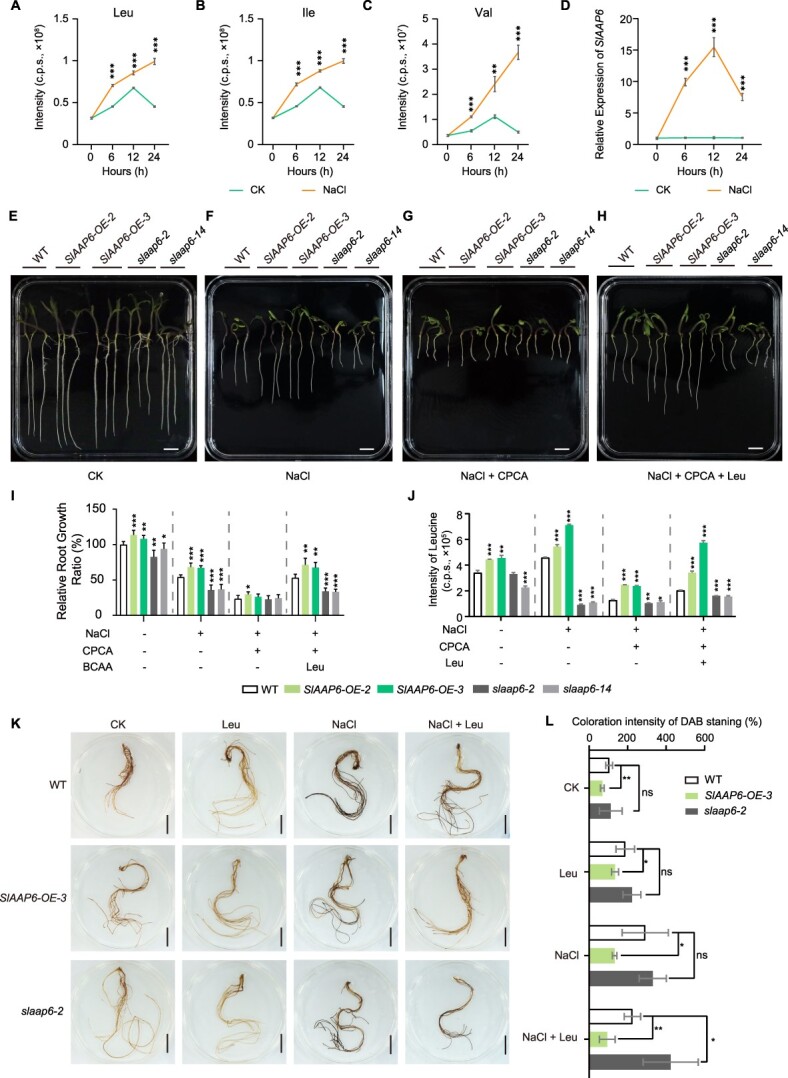
SlAAP6 enhanced tolerance to salinity stress via Leu transport. **A–C**: Leu (**A**), Ile (**B**), and Val (**C**) metabolite levels of tomato seedlings exposed to 150 mM NaCl for 0, 6, 12, and 24 h (*n* = 3). **D**: Relative expression of *SlAAP6* in tomato seedlings exposed to 150 mM NaCl for 0, 6, 12, and 24 h. **E–H**: Seven-day-old WT, *SlAAP6-OE* lines, and mutant seedlings grown on ½ MS medium (**E**) or medium supplemented with 150 mM NaCl (**F**), 150 mM NaCl and 0.5 mM CPCA (**G**), or 150 mM NaCl and 0.5 mM CPCA and 0.1 mM Leu (**H**) (*n* = 6). **I**: Bar plot of root length in WT, *SlAAP6-OE-2*, *SlAAP6-OE-3*, *slaap6-2*, and *slaap6-14* mutants grown on ½ MS medium (**E**) or medium supplemented with 150 mM NaCl (**F**), 150 mM NaCl and 0.5 mM CPCA (**G**), or 150 mM NaCl and 0.5 mM CPCA and 0.1 mM Leu (**H**) (*n* = 5). **J**: The abundance of endogenous Leu was quantified in WT and transgenic seedlings grown on ½ MS medium (**E**) or medium supplemented with 150 mM NaCl (**F**), 150 mM NaCl and 0.5 mM CPCA (**G**), or 150 mM NaCl and 0.5 mM CPCA and 0.1 mM Leu (**H**). **K–L**: H_2_O_2_ accumulation in roots was assessed through 3,3′-DAB staining (**K**), and the DAB staining intensity was quantified using ImageJ (**L**) (*n* = 5). Scale bar, 1 cm. All values are means ± SDs. Significant differences (Student's *t*-tests): ^*^P < 0.05; ^**^P < 0.01; ^***^P < 0.001.

Although BCAAs were proven as crucial metabolites for plant growth under saline environments [30], there is uncertainty about their transport mechanism under salt stress. To explore the accumulated pattern of BCAAs in tomato under salt stress, metabolite samples were collected from seedlings across four time points. Briefly, the content of Leu, Ile, and Val were accelerated accumulation during NaCl treatment (Fig. 4A–C). Simultaneously, the transcript levels of *SlAAP6* were examined to verify whether *SlAAP6* was induced expression during this progress. As expected, the expression of *SlAAP6* was upregulated prominently, implying that SlAAP6 plays an important role in salt stress (Fig. 4D). As SlAAP6 transports BCAAs in tomato, its response to salt stress was further examined. Compared to the WT, *SlAAP6-OE* lines showed notably longer roots, while *slaap6* mutants exhibited shorter roots, under non-salt stress conditions (Fig. 4E and I, and Fig. S8). When treated with 150 mM NaCl, *SlAAP6-OE* lines maintained the longest primary roots, whereas *slaap6* mutants produced the shortest roots (Fig. 4F). This suggests that *slaap6* mutants showed greater sensitivity to salt stress than either the WT or *SlAAP6-OE*. Whether or not *SlAAP6-OE* conferred salt tolerance was highly dependent on the BCAA content *in vivo*. To restrict endogenous BCAA biosynthesis in the NaCl treatments, 1,1-cyclopropanedicarboxylic acid (CPCA) was used. Blocking BCAA biosynthesis worsened seedling responses to NaCl that root elongation was significantly inhibited in the combined CPCA and NaCl treatment as compared to the NaCl treatment (Fig. 4G). In particular, the root length of WT, *SlAAP6-OE*, and *slaap6* mutants decreased by 56.70%, 56.54%, and 33.54%, respectively, when BCAA biosynthesis was restricted *in vivo* (Fig. 4G and I, and Fig. S8C). Thus, decreasing BCAA availability substantially reduced the salt tolerance of *SlAAP6-OE* lines. When exogenous BCAAs was supplied, normal root elongation was restored in WT and *SlAAP6-OE* under NaCl treatment, but not in *slaap6* mutants (Fig. 4H and Fig. S8). Strikingly, Leu, Ile, and Val showed different capacities to restore root elongation. Among them, exogenously supplied Leu caused a higher relative root growth ratio than Ile and Val application (Fig. 4H and Fig. S8). Similarly, Leu abundance was effectively decreased by CPCA treatment in all seedlings, but Leu uptake was restored in *SlAAP6-OE* lines (Fig. 4J). When exposed to a higher concentration of NaCl (200 mM), root length was further shortened in *slaap6* mutants, and could not be restored by the addition of exogenous Leu (Fig. S9). These findings suggest that BCAAs and their downstream metabolites promote tolerance to saline environments, dependent on the availability of amino acid transporters, and Leu promotes tomato root development and enhances salt tolerance.

Salt stress usually results in excess ROS accumulation, and amino acids have been shown to inhibit ROS accumulation under salt stress [55, 56]. Here, hydroponic cultures were used to examine the effects of *SlAAP6* on ROS elimination in the 200 mM NaCl treatment. The 3,3′-diaminobenzidine (DAB) and nitroblue tetrazolium (NBT) staining intensity of all transgenic lines and the WT was minimal in the control and 0.1 mM Leu treatments, but it increased significantly in the NaCl treatments (Fig. 4K–L and Fig. S10). Under saline conditions, only low levels of ROS accumulated in *SlAAP6-OE* lines and even less ROS accumulated following the application of 0.1 mM Leu (Fig. 4K–L and Fig. S10). Overall, these findings suggest that SlAAP6 transport of Leu repressed ROS accumulation in tomato roots, vitally improving seedling salt tolerance.

### BCAA addition effectively blocks H_2_O_2_ accumulation in tomato roots

**Figure 5:**
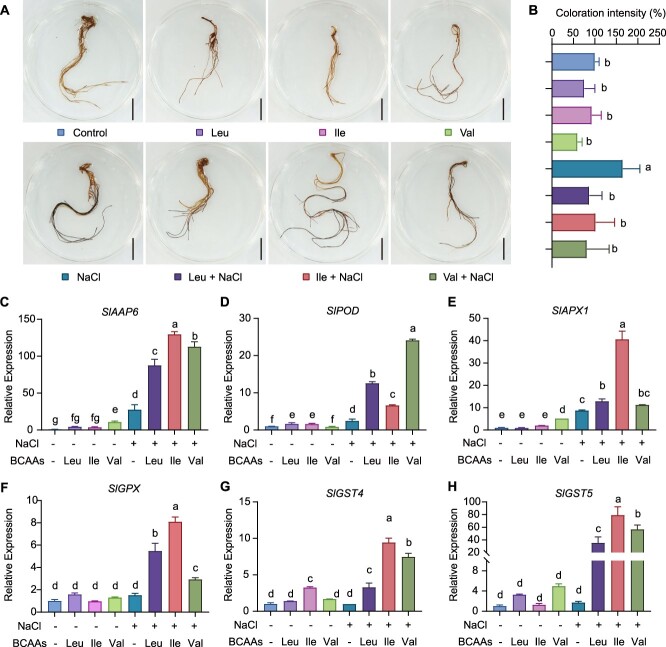
Exogenous BCAAs altered H_2_O_2_ accumulation and the expression of related genes in NaCl treatments. **A**: DAB staining of roots from tomato seedlings grown under normal condition (control), 0.1 mM Leu, 0.5 mM Ile, 0.5 mM Val, 200 mM NaCl, 0.1 mM Leu and 200 mM NaCl, 0.5 mM Ile and 200 mM NaCl, and 0.5 mM Val and 200 mM NaCl. Scale bar, 1 cm. **B**: Coloration intensity of DAB staining in roots depicted in (**A**) (*n* = 5). **C–H**: Relative expression of *SlAAP6* (**C**), *SlPOD* (**D**), *SlAPX1* (**E**), *SlGPX* (**F**), *SlGST4* (**G**) and *SlGST5* (**H**) in WT plants grown in hydroponic culture, as illustrated in (**A**). Different lowercase letters represented statistically significant differences among samples (*P* < 0.05).

The protective role of amino acids under salt stress is attributed to the induction of antioxidant enzyme activity [55, 56]. To determine the role of BCAAs in H_2_O_2_ desensitization in tomato roots under saline conditions, DAB staining was performed and the expression of H_2_O_2_-related enzymes was examined. No substantial differences in DAB staining were observed under normal conditions versus individual BCAA treatments; however, staining intensity was substantially higher in the 200 mM NaCl treatment versus the control (Fig. 5A and B). Compared to the pure NaCl treatment, the addition of Ile, Leu, or Val significantly reduced root staining under salt stress (Fig. 5A and B). In the expression analysis, H_2_O_2_ pathway-related enzyme production was evaluated for the BCAA and salt treatments. Interestingly, *SlAAP6* expression could be induced by NaCl application, as well as more strongly induced by BCAA application, implying that *SlAAP6* responded to salt stress (Fig. 5C). Peroxidase (*SlPOD*), a key enzyme catalyzing the degradation of H_2_O_2_, was highly expressed under salt stress as compared to control conditions (Fig. 5D). However, *SlPOD* showed higher expression patterns pronouncedly when BCAAs were added to the salt treatment (Fig. 5D). In addition, ascorbate peroxidase (*SlAPX1*) and glutathione S-transferase/peroxidase (*SlGST4*, *SlGST5*, and *SlGPX*) expression was enhanced under dual BCAA and NaCl treatment (Fig. 5E–H). Moreover, the activity of POD, APX, and GST was notably accumulated in BCAAs and salt stress (Fig. S11). Under salt stress, ROS quickly accumulated in tomato roots, but BCAA application significantly elevated H_2_O_2_ catabolism by inducing the expression of enzymes related to H_2_O_2_ degradation.

## Discussion

Although BCAAs are essential organic N sources for plant growth and key metabolites in plant stress responses, the mechanisms underlying BCAA accumulation in tomato remain elusive. Here, the BCAA content in 374 tomato accessions was assessed, and a single gene, *SlAAP6*, identified via mGWAS and functionally characterized as a major determinant of BCAA accumulation.

In tomato, the AAT-family member *SlAAP6* has been identified as one of at least 88 family members potentially involved in amino acid transportation during plant growth and development [35]. Loss of function in amino acid permease (AAP) genes results in the inhibition of amino acid transport, as well as alterations to amino acid concentrations within plant tissues [51]. Similar to OsAAP1 and OsAAP3 [57], SlAAP6 was located at the plasma membrane, resulting in amino acids transport among cells. Compared to described AAPs like AtAAP6, GmAAP6, OsAAP6, and StAAP1 [47, 49, 50], SlAAP6 may increase the availability of free amino acids by mediating neutral and basic amino acid transport, as suggested by the following findings. First, transport experiments with yeast strain and frog oocytes support that SlAAP6 is a high-affinity transporter involved in import of amino acids including Leu and Ile. Second, *SlAAP6-OE* lines showed elevated neutral and basic amino acid accumulation, while no difference (or even lower concentrations) was observed in *slaap6* mutants. Considering that changes in the concentration of a single amino acid in plants can affect the homeostasis of other amino acids through catabolism [58], the classes of amino acids transported by SlAAP6 in tomato are different from those transported in yeast cells. Crucially, knockout of SlAAP6 in tomato reduce the absorption of ^15^N-labeled BCAAs in roots and their translocation to shoots. Third, the total free amino acid content in both shoots and roots increased in *SlAAP6-OE*, but decreased significantly in *slaap6*, suggesting the inhibition of amino acid transport to shoots. Thus, *SlAAP6* plays a critical role in the root uptake and transport of multiple amino acids to aerial plant parts in tomato. However, further study is needed to elucidate the specific role of SlAAP6 in altering amino acid levels in pollen, fruits, and seeds.

In this study, increasing *SlAAP6* expression in tomato promoted seedling growth and development. Compared to WT plants, *SlAAP6-OE* seedling biomass was elevated in both roots and shoots, while *slaap6* seedlings were stunted under normal hydroponic growth conditions. It was established that AAPs facilitated amino acid content to elevate nitrogen content, hence directly improves the development of plants [33, 49]. Given the difference in total free amino acid abundance and total nitrogen content between *SlAAP6-OE* and *slaap6* seedlings, SlAAP6 likely plays a crucial role in original nitrogen accumulation and growth and development in tomato. As a positive regulator of BCAA content, *SlAAP6* greatly enhanced root growth when individual BCAAs were supplemented. There are likely several factors underlying the root phenotypic differences observed for *SlAAP6-OE* and *slaap6*. First, differences in root growth between *SlAAP6-OE* and *slaap6* mutants may have been driven by limitations in BCAA uptake. For example, restrictions on amino acid uptake in the roots of *oslht1* mutants also decrease the import of “toxic” lysine, thereby preserving plant growth [53, 59]. Studies in cucumber and rice have shown that concentration gradients of amino acids may cause nitrogen stress, resulting in fluctuations in root growth [53, 54, 60]. Here, the supply of a high BCAA concentration also resulted in a reduced growth phenotype in WT, while the *SlAAP6-OE* and *slaap6* lines exhibited greater tolerance and sensitivity to BCAAs, respectively. Due to the allocation of amino acids from root to shoot, *SlAAP6-OE* lines transported more BCAAs to the aerial parts, thereby mitigating limited growth of root, whereas the lack of difference in root growth between *SlAAP6-OE* and *slaap6* lines under 0.1 mM Ile treatment may be attributed to the redundant functional impact of other AAPs in tomato. Moreover, BCAA metabolism affects both cell proliferation and cell expansion processes during root development [23], and overaccumulation of BCAAs induces cell proliferation [61]. In this study, the expression of genes related to root growth and cell proliferation was upregulated in *SlAAP6-OE*, but unaffected in *slaap6* seedlings. Therefore, differences in seedling growth between *SlAAP6-OE* and *slaap6* mutants could be the result of differences in nutrient uptake.

Although these findings suggest that BCAAs accumulate in tomato seedlings under NaCl conditions similar to other species [30], the detailed mechanisms of BCAA translocation are not yet understood. Since the expression of *SlAAP6* rapidly responds to saline treatment, the involvement of SlAAP6 in enhancing salt tolerance via BCAA transport was demonstrated. In this study, root growth in *slaap6* mutants was highly sensitive to the medium NaCl concentration, while *SlAAP6-OE* lines showed elevated salinity tolerance compared with the WT. Thereby, BCAA transport is a necessary strategy that helps tomato cope with salt stress. The sensitivity of *slaap6* mutants to salt was primarily attributed to reduced levels of endogenous BCAAs in plant roots, whereas *SlAAP6-OE* lines exhibited higher BCAA levels, contributing to their salt tolerance. When endogenous BCAA synthesis was disrupted, the salt tolerance of *SlAAP6-OE* plants was suppressed, indicating that BCAAs confer salt tolerance in tomato, dependent on the availability of SlAAP6. Similar to *ProT1*, which modulated proline flux in other species cells to enhance salt tolerance [43, 45], *SlAAP6* rapidly responded to NaCl stress, sustaining essential BCAA uptake in tomato roots. In *Arabidopsis* and rice, BCAA homeostasis impacts salinity tolerance, and decreasing endogenous BCAA levels produced salt hypersensitivity responses in roots [29], while elevated BCAA contents contribute to ROS detoxification [30]. ROS serve as an early signal in response to ER stress caused by misfolded or unfolded proteins accumulating in the ER under abiotic stimuli, such as salt stress [62]. In response to ER stress under saline stimuli, the expression of genes involved in the ER stress pathway is upregulated, and these proteins must be located in or transported to the ER [63]. Given that SlAAP6-GFP signals are also in the ER, we predicted that SlAAP6 responds to ER stress caused by salt stress and positively eliminates ROS by modulating relevant signals. Under saline stress, ROS quickly accumulated in tomato roots, but BCAA application significantly elevated H_2_O_2_ metabolism by inducing the expression of enzymes related to H_2_O_2_ degradation. Based on these findings, *SlAAP6* likely plays a crucial role in BCAA transport, contributing to tomato salt tolerance.

**Figure 6:**
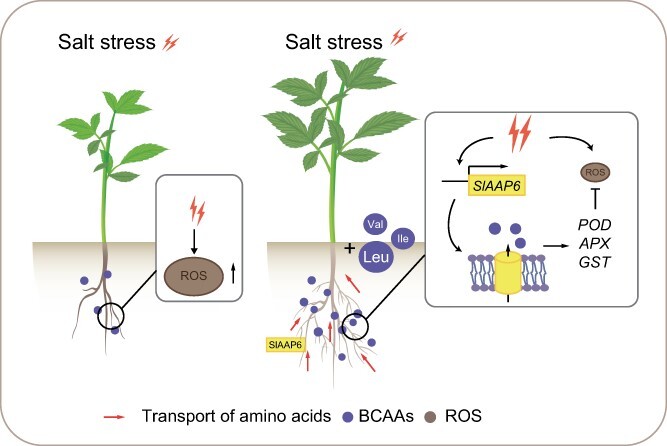
A proposed model for the role of *SlAAP6* in the salt stress response. The roots of tomato exhibit restricted growth and higher accumulation of ROS under salt stress. However, BCAAs are rapidly accumulated *in vivo* under NaCl treatment. Meanwhile, *SlAAP6* responds to salt stress and contributes to increasing the levels of BCAAs in tomato roots. When exogenous BCAAs are supplied in NaCl conditions, root growth is rescued and ROS levels decrease significantly, attributed to the uptake and transposition of BCAAs through SlAAP6. BCAAs serve as nonenzymatic antioxidants that promote the expression of *POD* (peroxidase), *APX* (ascorbate peroxidase), and *GST* (glutathione S-transferase), resulting in the elimination of excess ROS. Induced expression of *SlAAP6* accelerates BCAAs accumulation, thus maintaining optimal ROS homeostasis to sustain tomato growth under saline environments.

In summary, SlAAP6 modulated the uptake and transport of a range of amino acids and was a positive regulator of tomato growth and development. Increasing the expression of *SlAAP6* contributed to enhanced BCAA absorption in plant roots, leading to root cell proliferation. Moreover, SlAAP6 promoted salt tolerance through BCAA transport. Therefore, *SlAAP6* represents a useful candidate gene for tomato genetic engineering, allowing for the simultaneous improvement of amino acid availability, growth enhancement, and increased resistance to salt stress (Fig. 6).

## Materials and methods

### Plant materials and culture conditions

The tomato cultivar MicroTom was used as the WT in this study. All tomato seedlings were grown under controlled conditions with a 16:8 h light-to-dark regime at 25°C and 60% humidity. Seeds were sterilized for 12 min in a 0.5% (w/v) sodium dichloroisocyanurate solution, washed with sterilized ultrapure water, then cultured on half strength Murashige and Skoog medium (½ MS) at 25°C in the dark for 2 days. After the 2 days, seeds were moved into an explant culture chamber with a photoperiod of 16:8 h light-to-dark for germination. The 7-day-old seedlings were prepared for BCAA and salinity treatments to examine growth suppression.

### Generation of transgenic plants and growth treatments

The *SlAAP6* CDS was cloned from MicroTom into pDONR207 using Gateway (Invitrogen) technology, and then transferred to the pBI121 vector to generate *35S::SlAAP6* constructs [64]. The design of the sgRNAs and generation of the pTX041_*SlAAP6* constructs followed previously published protocols [64]. To generate *ProSlAAP6::GUS* constructs, a 2000-bp *SlAAP6* promoter fragment from the MicroTom genome was inserted before the β-glucuronidase (GUS) reporter gene in pHGWFS7.0 vector. The stable transformation of binary vectors into MicroTom was achieved through *Agrobacterium tumefaciens* (LBA4404) infection, as previously described [7]. All explants and transgenic seedlings were selected on media containing kanamycin. Real-time quantitative reverse-transcription PCR (qRT-PCR) and DNA sequencing were employed to identify transgenic plants for *SlAAP6*-overexpression lines and knockout mutants, respectively. The presence of *ProSlAAP6::GUS* constructs in tomato was confirmed utilizing GUS staining and PCR analysis. Primers used in this study for the generation of constructs and gene identification are listed in Table S3.

To evaluate the growth of transgenic seedlings, 7-day-old *SlAAP6-OE* and *slaap6* mutant seedlings were transferred from solid ½ MS medium with kanamycin to liquid MS medium, then cultured for 14 days. Seedling weight and the total free amino acid content were compared between transgenic seedlings and the WT.

To identify individual amino acids transported by SlAAP6, 21-day-old seedlings samples were collected from WT, *SlAAP6-OE*, and *slaap6* mutant seedlings and incubated with a mixture of all 20 amino acids (25 mM per amino acid) for 6 h [50]. For uptake studies with ^15^N-labeled BCAAs, 21-day-old seedlings were treated with 0.1 mM concentration for 6 h as described [53]. ^15^N-labeled BCAAs were used in this paper included ^15^N-leucine (atom% ^15^N: 99%), ^15^N-Isoleucine (atom% ^15^N: 98%), and ^15^N-valine (atom% ^15^N: 98%).

For exogenous BCAA treatments, similar 7-day-old seedlings were selected and grown in N-deficient ½ MS media with several concentrations (0, 0.1, 0.5, and 1 mM) of Leu, Ile, or Val as the sole N source for 7 days. Root length was measured with a ruler.

### Genome-wide association analysis and phylogenetic analysis

A metabolite genome-wide association study (mGWAS) was performed based on the BCAA data for the 374 tomato accessions as described previously [46]. A description of the 374 tomato varieties is provided in Table S4.

Full-length protein sequences of the amino acid permeases (AAPs) used in this study were obtained from UniProt (https://www.uniprot.org/). A phylogenetic tree was constructed in MEGAX using the neighbor-joining method; bootstrap values (one thousand replicates) were calculated and illustrated on the tree; all the protein sequences used in this study are listed in Table S5. A phylogenetic analysis of AAP6 and AAP6-like proteins from MEGAX was imported into GeneDoc to display the aligned amino acid sequences. The transmembrane domains of SlAAP6 were predicted using the DeepTMHMM website (https://dtu.biolib.com/DeepTMHMM). cDNA and protein sequence data generated for this study can be obtained from the Sol Genomics Network (https://solgenomics.net/) and UniProt (https://www.uniprot.org/) databases.

### Expression patterns of *SlAAP6* and RNA extraction

To examine *SlAAP6* expression pattern, RNA was extracted from eight different tissues: stems, leaves, flowers, immature green fruits, breaker fruits, ripe fruits, seeds, and roots.

Tomato RNA samples were extracted using a TransZol Up reagent kit (TransGen Biotech, Beijing). To obtain cDNA, reverse transcription of RNA samples was used; 2-μg samples were suspended in 20 μl of ToloScript All-in-One RT EasyMix for qPCR (TOLOBIO, Shanghai). Transcript abundance was quantified by utilizing 2 × Q3 SYBR qPCR Master Mix (TOLOBIO) on the QuantStudio™ 7 Pro Real-Time PCR system (Applied Biosystems, USA). The qRT-PCR results were normalized using the reference gene *SlACT2* (*Solyc11g005330*). Relative expression levels are presented as mean normalized transcript levels as calculated via the comparative cycle threshold method (2^−ΔΔCt^). The gene-specific primers used for qRT-PCR are listed in Table S3.

### GUS staining analysis for SlAAP6

β-Glucuronidase (GUS) staining was performed in *ProSlAAP6*::*GUS* transgenic lines. Whole tomato seedlings were obtained and stained with GUS dye solution at 37°C overnight. The stained samples were then transferred to 95% ethanol and incubated at 37°C for 24 h. The stained samples were observed using a stereomicroscope (Carl Zeiss Microscopy Axio Zoom.V16).

### Subcellular localization of SlAAP6

The full-length CDS of *SlAAP6* was transferred into a pK7WGF2 vector fused with green fluorescent protein (GFP) using Gateway technology to generate 35S::SlAAP6-GFP fusion constructs. Fusion vector and marker protein vector were mixed and cotransformed into prepared protoplasts of tobacco (*N. benthamiana*) leaves [50]. And the constructs were transiently cotransformed into tobacco leaves using *A. tumefaciens* (strain GV3101 pSoup-p19). Fluorescence signals of the SlAAP6-GFP fusion proteins, plasma membrane [65], and ER markers [66] in tobacco were observed using confocal laser scanning microscopy (Leica Microsystem LAS AF) [50].

### Functional complementation of SlAAP6 in yeast

The full-length CDS of *AtLHT1* and *SlAAP6* were fused into a pESC-URA vector using Gateway technology as described above (for other experiments). The *AtLHT1* and *SlAAP6* constructs, along with the pESC-URA vector (empty vector, EV), were transformed into the *Saccharomyces cerevisiae* mutant strain 22Δ10α [52]. The genotype of yeast mutant strain 22Δ10α (*MATα, gap1-1, put4-1, uga4-1, can1::HisG lyp1/alp1::HisG, hip1::HisG, dip5::HisG, gnp1Δ, agp1Δ, ura3-1*) was described as reported [67]. The yeast WT strain 23344c was used as a growth control. The incubation media and growth conditions for the yeast cells followed Guo *et al.* [53]. Yeast cells (3 μl of solution) were grown on yeast nitrogen base media with a single N source, either 1 mM of a single amino acid or (NH_4_)_2_SO_4_. All plates were cultured at 28°C for 48–72 h to allow for yeast growth. Furthermore, the 22Δ10α transformed by EV and *SlAAP6* were use for 0.5 mM ^15^N-labeled BCAAs feeding study as described as Guo *et al.* [53].

### Expression in *Xenopus laevis* oocytes

The expression of SlAAP6 in African clawed frog *X. laevis* oocytes were perform as reported study [39, 68]. *SlAAP6* cRNA was synthesized from the *Not* I-linearized *SlAAP6*-pGH19 construct by *in vitro* transcription using the mMACHINE® high yield capped RNA kit. The oocytes were then injected with 45 nl of *SlAAP6* cRNA (1 ng nl^−1^ in RNase-free ddH_2_O) or 45 nl of RNase-free ddH_2_O, which was the negative control by using Nanoject III (Auto-Nanoliter Injector, Drummond Scientific Company) and incubated in ND96 for 24 h at 16°C. To conduct ^15^N-BCAA influx analysis, oocytes were cultured in a bath solution (ND96 medium) and incubated in the bath solution containing 50 μM ^15^N-leucine (atom% ^15^N: 99%), ^15^N-Isoleucine (atom% ^15^N: 98%), or ^15^N-valine (atom% ^15^N: 98%), respectively, for 20 min at 16°C. In this experiment, 15 oocytes were examined in triplicate. Then the oocytes were washed several times with ddH_2_O, ground with 200-μl 50% methanol (v/v), and measured through LC–MS.

### Salinity stress treatments and physiological measurements

To determine metabolite levels of BCAAs and transcript levels of *SlAAP6* in tomato seedlings under salt stress, similarly sized 21-day-old seedlings were incubated with 150 mM NaCl liquid MS solution or liquid MS solution (control condition). And the root samples were collected from control condition at 0 h and 150 mM NaCl treatment for 6, 12, 24 h.

To examine how BCAAs respond to salinity stress, root-growth assays were carried out using T2 or T3 seedlings from *SlAAP6OE-2*, *SlAAP6OE-3*, *slaap6-2*, and *slaap6-14*. Seedlings were grown on ½ MS and exposed to different salt stress treatments: 150 mM NaCl; 200 mM NaCl; 150 mM NaCl and 0.5 mM CPCA; 150 mM NaCl and 0.5 mM CPCA with an additional 0.1 mM Leu; or 200 mM NaCl and 0.1 mM Leu. For the control, ½ MS medium was used. Assays took place under a 16:8 h light-to-dark regime at 25°C for 5 days. Root length was measured with a ruler.

To test how *SlAAP6* expression affects ROS accumulation under salt stress conditions, similar-sized 21-day-old seedling roots were incubated in ddH_2_O with 200 mM NaCl and 0.1 mM Leu, or 200 mM NaCl and 0.1 mM BCAA, for 12 h. Root samples were washed with ddH_2_O before staining with 3,3′-DAB and NBT [56].

To explore the effects of exogenous BCAA addition on H_2_O_2_-related enzyme transcript levels under saline conditions, seven BCAA treatments were applied to 21-day-old WT seedlings for 12 h. The treatment solutions consisted of 200 mM NaCl alone, a single BCAA, or a mixture of 200 mM NaCl and a single BCAA. After treatment, a part of the roots from each seedling was stained with DAB, while another section was collected using liquid nitrogen for later RNA extraction.

After staining, photos of the root samples were taken and ROS quantification was performed in ImageJ (https://imagej.nih.gov/ij/). Quantification steps were conducted as described as Gonorazky *et al.* [69].

The enzyme contents and the activities of POD (Kit No. BC0090), APX (Kit No. BC0220), and GST (Kit No. BC0350) in roots were measured according to the manufacturer's instructions (Solarbio, China).

### Total nitrogen analyses

T2 or T3 seedlings from *SlAAP6OE-2*, *SlAAP6OE-3*, *slaap6-2*, and *slaap6-14* were grown under liquid MS medium for 30 days. The whole seedlings were collected to determine total nitrogen content using the Kjeldahl method [33].

### Amino acid content detection in tomato

The total free amino acid content was measured using ninhydrin colorimetry as described by Yao *et al.* [54]. For the individual amino acid and ^15^N-BCAAs analyses, the entire tomato plant samples were finely ground into powder at 30 Hz after freezing with liquid nitrogen. For each sample, 0.1 g of the powder was weighed and 1 ml of 70% methanol (v/v) added for extraction. The samples were vortexed three times at 10-min intervals to ensure even mixing, then extracted at 4°C for 10 h and centrifuged at 13000*g* for 15 min. The supernatant was filtered through a 0.22-μm organic filter prior to measurement by LC–MS. Chromatographic and mass spectrometric parameters were set following Guo *et al.* [70]. In this study, metabolic signals were processed using the method of relative quantification, which analyzed the metabolic signal peak area of each extracted ion chromatogram in samples. Metabolite spectral data were analyzed in MultiQuant 3.0.3; peak area integration was performed on the mass spectrum peaks of all metabolites to obtain the metabolite content for each sample. The metabolite retention time and peak shape information were also utilized to ensure the accuracy of the measurements. A summary of the metabolome profiling results for each amino acid is provided in Table S6.

### Statistical analysis

All data plotting and statistical analyses were performed in GraphPad Prism 9.0 (https://www.graphpad.com/). Data are generally presented as means ± SDs (standard deviations), and significant differences were identified using Student *t*-tests or one-way ANOVAs followed by Duncan tests, as implemented in SPSS Statistics 19 (SPSS Inc., Chicago, IL).

### Accession numbers

*SlACT2* (Solyc11g005330), *SlAAP6* (Solyc04g077050), *SlSHR* (Solyc02g092370), *SlRPL11C* (Solyc02g086240), *SlCycB1;2* (Solyc10g080950), *SlCycB1* (Solyc10g078330), *SlCDKA1* (Solyc08g066330), *SlPOD* (Solyc11g018800), *SlGPX* (Solyc07g056480), *SlAPX1* (Solyc06g005160), *SlGST4* (Solyc09g011520), *SlGST5* (Solyc09g011540).

## Supplementary Material

Web_Material_uhae286
