# Supplementary material for: The amino acid permease SlAAP6 contributes to tomato growth and salt tolerance by mediating branched-chain amino acid transport
Source: Hortic Res. 2024 Oct 11;12(1):uhae286. doi: 10.1093/hr/uhae286 (PMC11775608; doi:10.1093/hr/uhae286)
Supplement: Web_Material_uhae286 [file web_material_uhae286.zip › 5. Supplementary Figures.pdf]

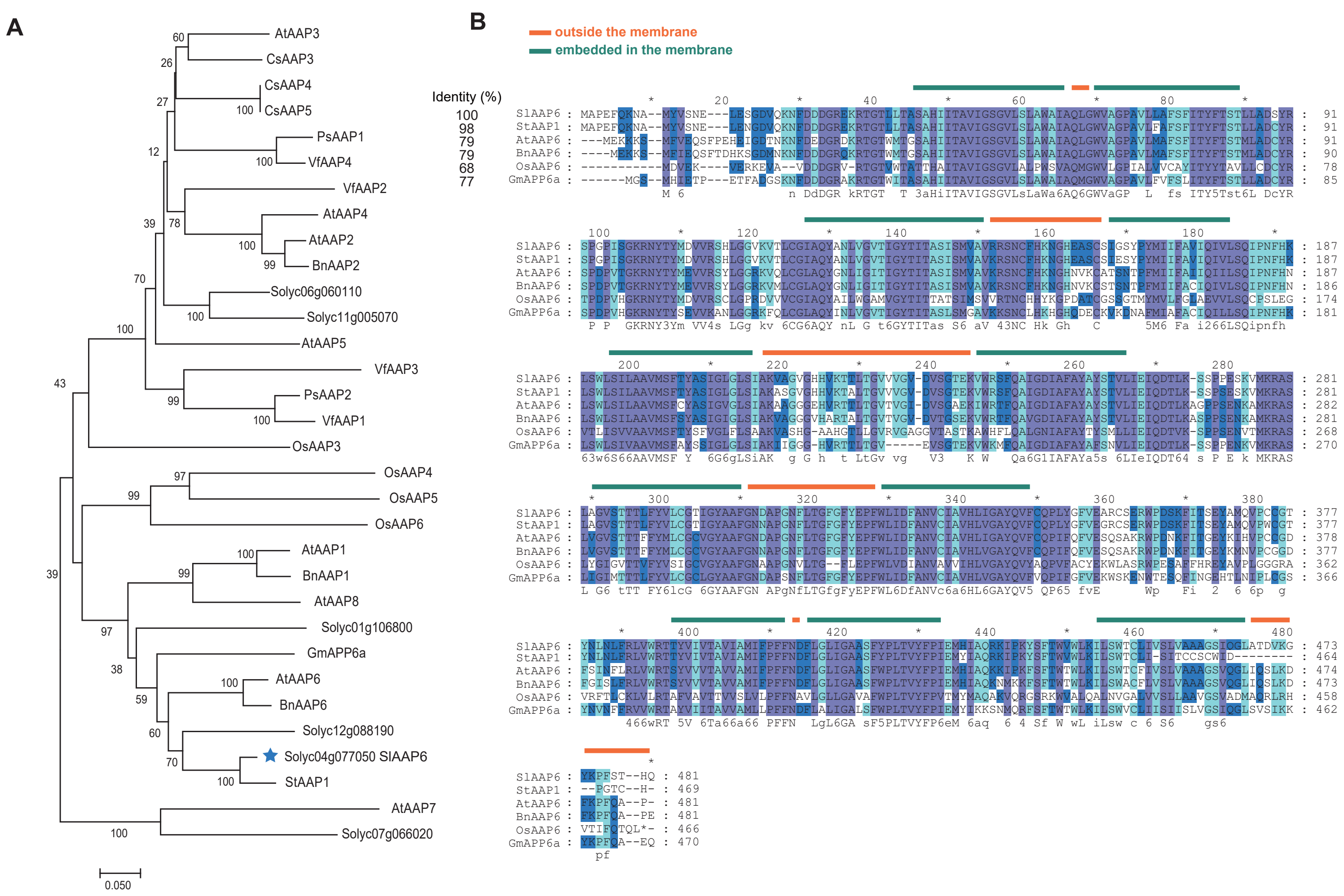

Figure S1 Phylogenetic analysis of SlAAP6.

A: Phylogenetic analysis of the SlAAP6 protein and other AAP proteins from *Arabidopsis thaliana*, *Brassica napus*, *Cucumis sativus*, *Glycine max*, *Oryza sativa*, *Pisum sativum*, *Solanum lycopersicum*, *Solanum tuberosum*, and *Vicia faba*. **B**: Alignment of the SlAAP6 protein sequence with homologous AAP6 or AAP6-like proteins performed using MEGA and GeneDoc. Orange and green lines represent outside and inner domains predicted by DeepTMHMM.

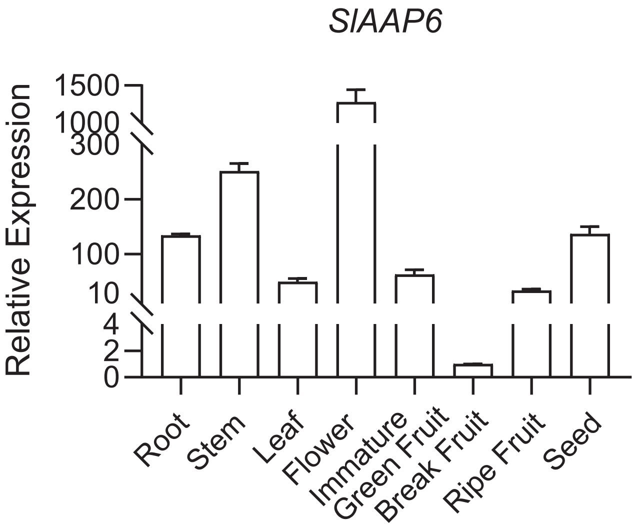

Figure S2 Tissue expression patterns of *SlAAP6*.

Real-time fluorescence quantification of organ-specific *SlAAP6* expression in tomato seedlings. The values are means ± SDs (n=3).

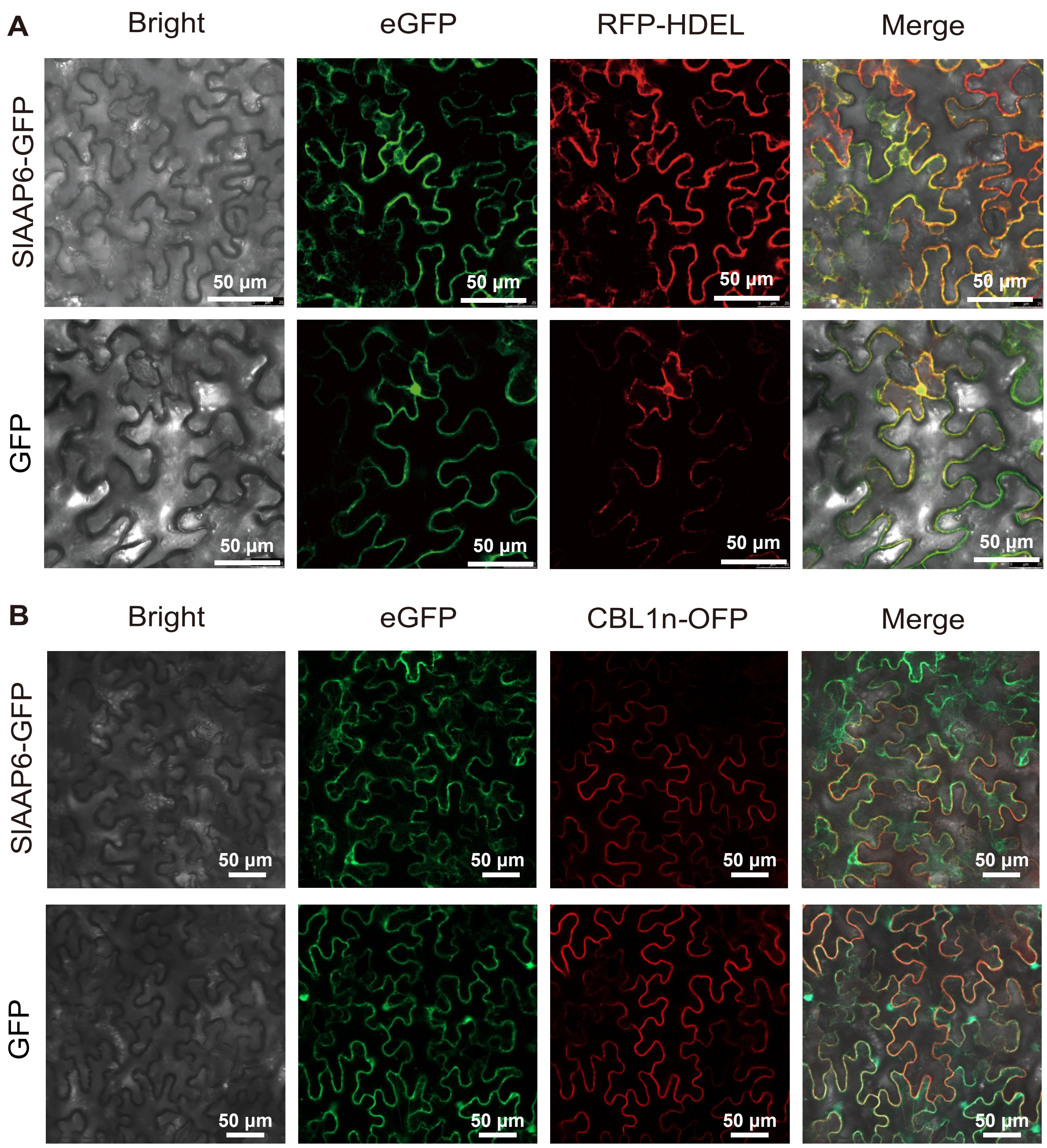

Figure S3 Subcellular localization analysis of SlAAP6 in tobacco leaves.

Transient expression of SlAAP6-GFP fusion protein in transfected tobacco leaves with ER marker (RFP-HDEL) (**A**) and plasma membrane marker (RFP-CBL) (**B**).

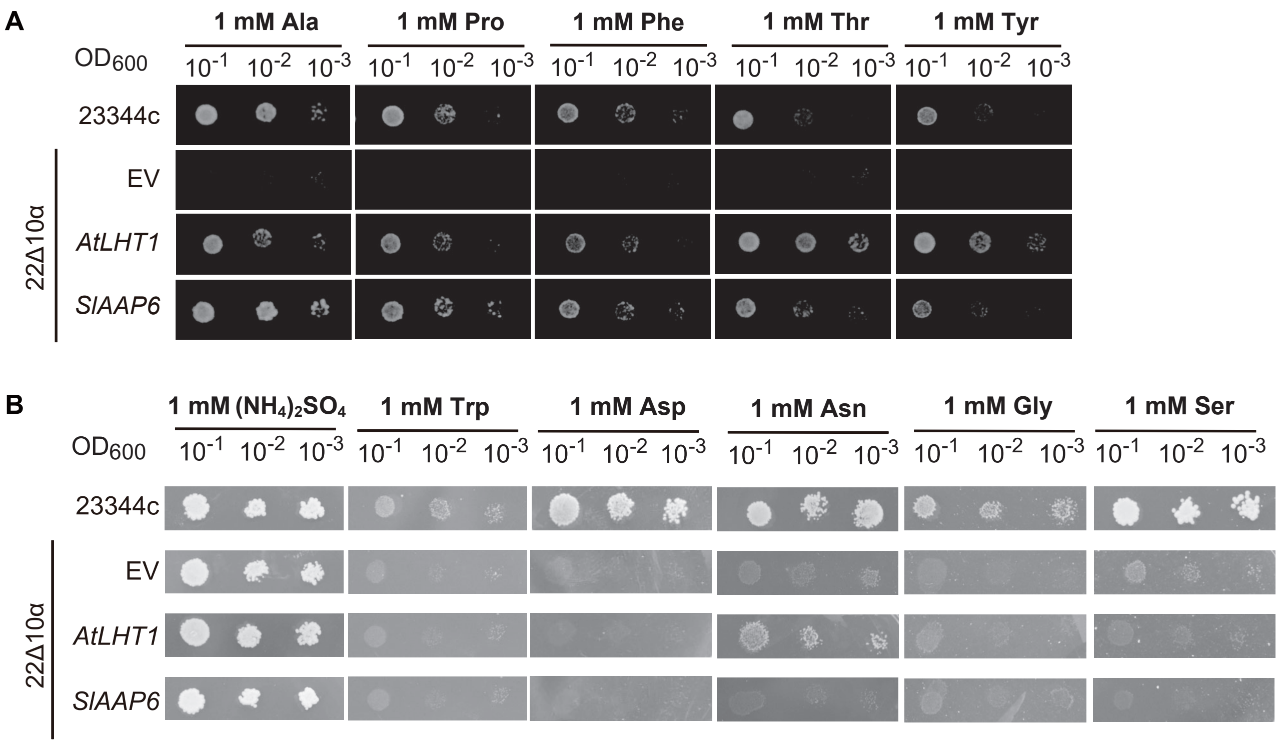

Figure S4 Heterologous expression of *SlAAP6* in yeast.

A: Heterologous expression of *SlAAP6* in yeast strain 22Δ10α grown on 1 mM amino acids, including alanine, proline, phenylalanine, threonine, and tyrosine. **B**: Heterologous expression of *SlAAP6* in yeast strain 22Δ10α is unable to grow on 1 mM amino acids, including tryptophan, aspartic acid, asparagine, glycine, and serine.

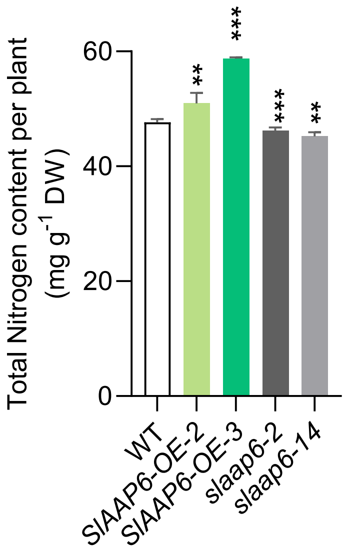

Figure S5 The total nitrogen contents of whole seedlings were detected from WT and *SlAAP6* transgenic lines.

All values are means ± SDs (n=3). Significant differences (Student's t-tests): **p* <0.05; ***p* <0.01; ****p* <0.001.

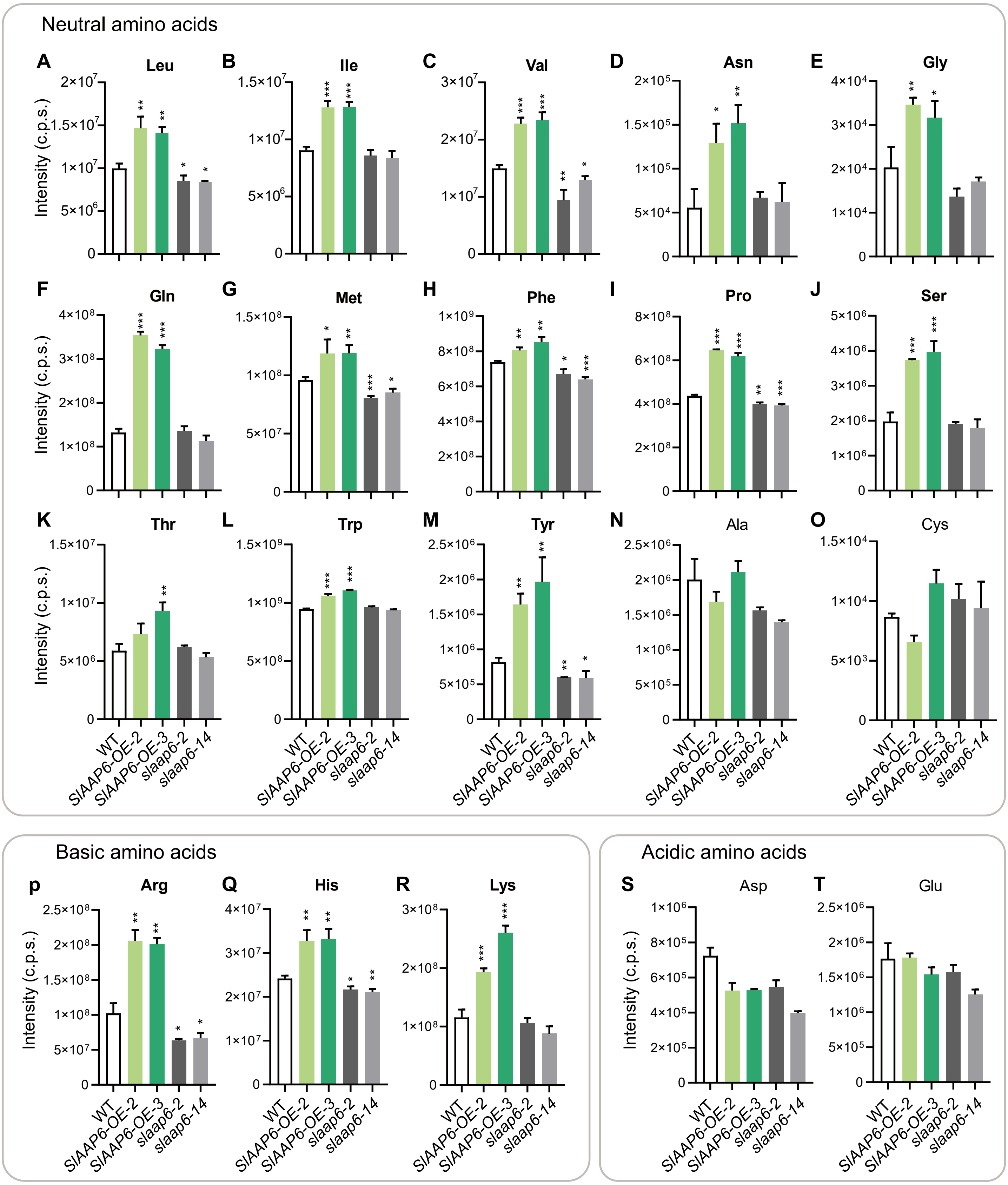

Figure S6 The amino acid composition of 21-day-old seedlings from WT and *SlAAP6* transgenic lines as analyzed by LC-MS.

Individual amino acids were classified as neutral amino acids (**A-O**), basic amino acids (**P-R**), or acidic amino acids (**S** and **T**). **A**: leucine, **B**: isoleucine, **C**: valine, **D**: asparagine, **E**: glycine, **F**: glutamine, **G**: methionine, **H**: phenylalanine, **I**: proline, **J**: serine, **K**: threonine, **L**: tryptophan, **M**: tyrosine, **N**: alanine, **O**: cysteine, **P**: arginine, **Q**: histidine, **R**: lysine, **S**: aspartic acid, **T**: glutamic acid. Amino acids transported by SlAAP6 are shown in bold (n=3). All values are means ± SDs. Significant differences (Student's *t*-tests): $*p < 0.05$; $**p < 0.01$; $***p < 0.001$.

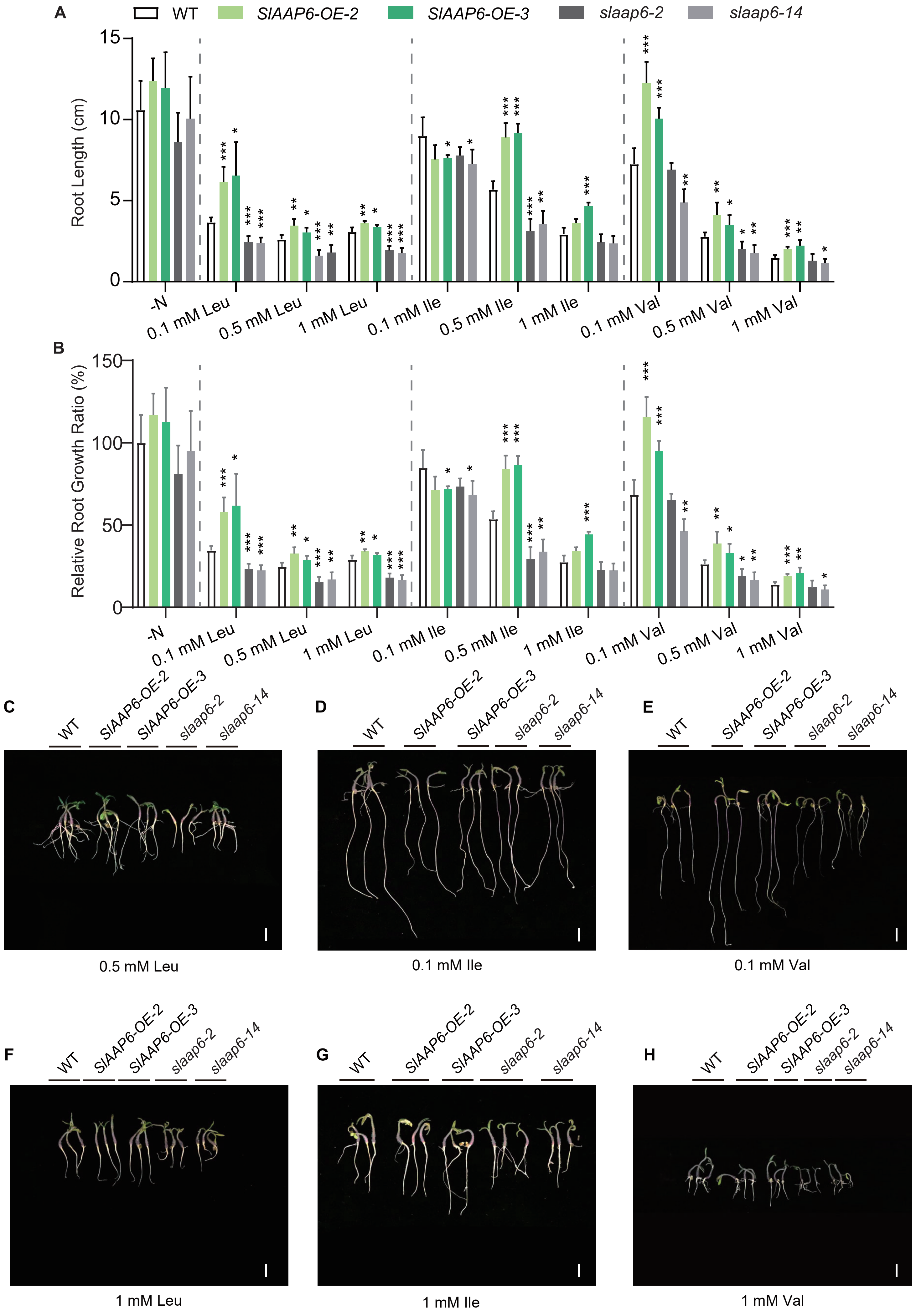
A
WT
SlAAP6-OE-2
SlAAP6-OE-3
slaap6-2
slaap6-14
Root Length (cm)
-N
0.1 mM Leu
0.5 mM Leu
1 mM Leu
0.1 mM Ile
0.5 mM Ile
1 mM Ile
0.1 mM Val
0.5 mM Val
1 mM Val
B
Relative Root Growth Ratio (%)
C
0.5 mM Leu
D
0.1 mM Ile
E
0.1 mM Val
F
1 mM Leu
G
1 mM Ile
H
1 mM Val

Figure S7 Root growth of WT and *SlAAP6* transgenic lines for different BCAA treatments.

Root length (**A**) and relative root growth ratio (**B**) comparison of WT, *SlAAP6-OE-2*, *SlAAP6-OE-3*, *slaap6-2*, and *slaap6-14* lines (n=5) and root phenotypes of sample plants grown on 0.5 mM Leu (**C**), 0.5 mM Ile (**D**), 0.5 mM Val (**E**), 1 mM Leu (**F**), 1 mM Ile (**G**), or 1 mM Val (**H**). Scales bars represent 1 cm. All values are means ± SDs. Significant differences (Student's *t*-tests): *p <0.05; **p <0.01; ***p <0.001.

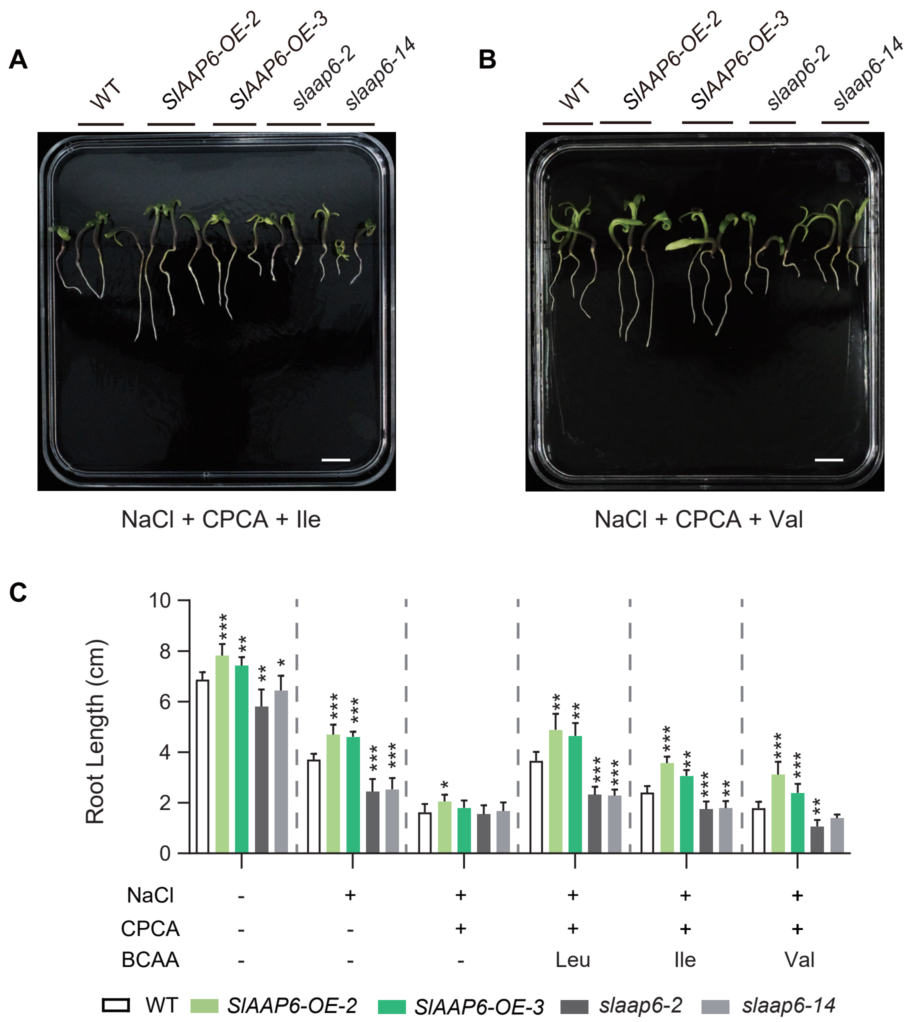

Figure S8 Root length comparison of *SlAAP6* transgenic seedlings and WT under salt stress with individual exogenous BCAA application.

A-B: Seven-day-old WT, *SlAAP6-OE* lines, and mutant seedlings grown on ½ MS medium with 150 mM NaCl and 0.5 mM CPCA and 0.1mM Ile (**A**), or 150 mM NaCl and 0.5 mM CPCA and 0.1mM Val (**B**) (n=6). **C**: Bar plot of root length in WT, *SlAAP6-OE-2*, *SlAAP6-OE-3*, *slaap6-2*, and *slaap6-14* mutants grown on ½ MS medium or medium supplemented with 150 mM NaCl, 150 mM NaCl and 0.5 mM CPCA, 150 mM NaCl and 0.5 mM CPCA and 0.1mM Leu, 150 mM NaCl and 0.5 mM CPCA and 0.1mM Ile (**A**), or 150 mM NaCl and 0.5 mM CPCA and 0.1mM Val (**B**) (n=5). Scale bar, 1 cm. All values are means ± SDs. Significant differences (Student's *t*-tests): *p <0.05; **p<0.01; ***p<0.001.

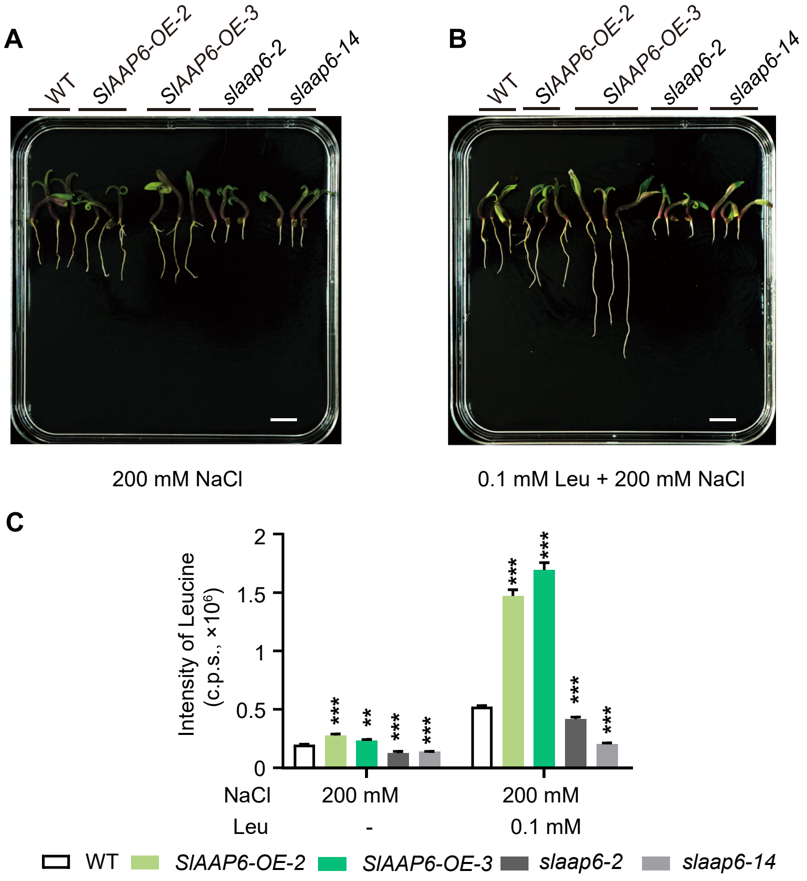

Figure S9 *SlAAP6* knockouts were sensitive to high salt concentrations.

Seven-day-old WT and *SlAAP6* transgenic seedlings were grown on media supplemented with 200 mM NaCl (**A**) or 200 mM NaCl and 0.1mM Leu (**B**). The relative abundance of endogenous Leu (**C**) was quantified in seedlings grown in the 200 mM NaCl (**A**), and 200 mM NaCl and 0.1mM Leu (**B**) treatments. All values are means ± SDs. Significant differences (Student's *t*-tests): **p* <0.05; ***p* <0.01; ****p* <0.001.

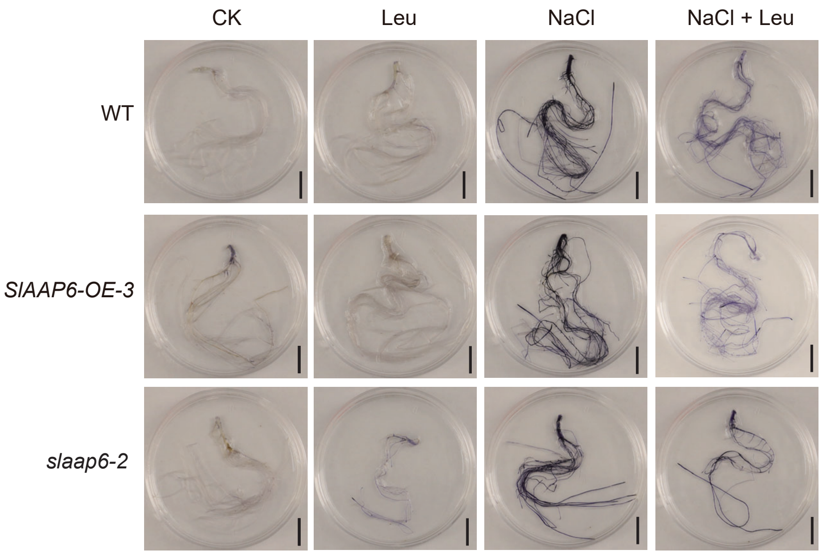

Figure S10 Superoxide anion in roots was assessed through nitroblue tetrazolium (NBT) staining.

Scale bar, 1 cm.

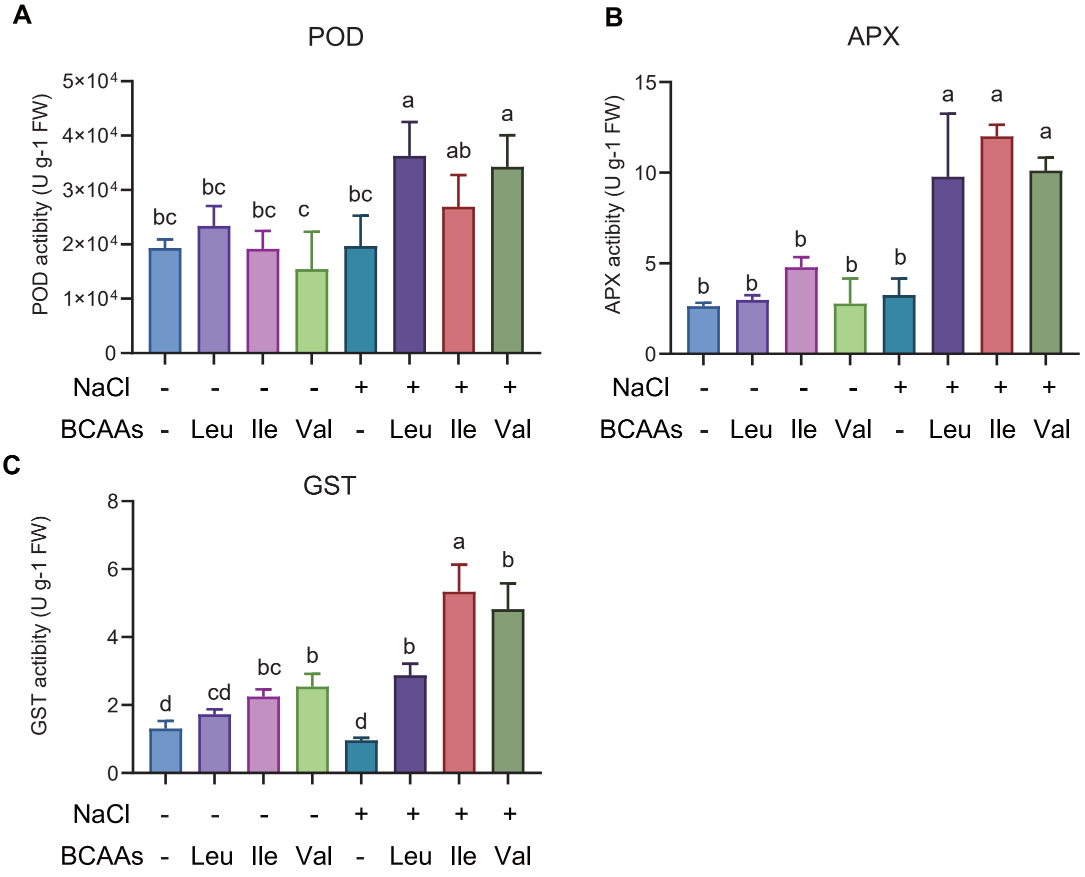

Figure S11 The enzyme activity of POD, APX, and GST in tomato roots under exogenous BCAAs in NaCl treatments.

A-C: The enzyme activity of POD (**A**), APX (**B**), and GST (**C**) in WT plants grown in hydroponic culture, as illustrated in liquid MS (control), 0.1 mM Leu, 0.5 mM Ile, 0.5 mM Val, 200 mM NaCl, 0.1 mM Leu and 200 mM NaCl, 0.5 mM Ile and 200 mM NaCl, and 0.5 mM Val and 200 mM NaCl. Different lowercase letters represented statistically significant differences among samples ($p < 0.05$).
